# A coarse-graining, ultrametric approach to resolve the phylogeny of prokaryotic strains with frequent homologous recombination

**DOI:** 10.1186/s12862-020-01616-5

**Published:** 2020-05-07

**Authors:** Tin Yau Pang

**Affiliations:** Computational Cell Biology, Heinrich Heine University, 40225 Düsseldorf, Germany

**Keywords:** Phylogenetics, Homologous recombination, Ultrametric tree

## Abstract

**Background:**

A frequent event in the evolution of prokaryotic genomes is homologous recombination, where a foreign DNA stretch replaces a genomic region similar in sequence. Recombination can affect the relative position of two genomes in a phylogenetic reconstruction in two different ways: (i) one genome can recombine with a DNA stretch that is similar to the other genome, thereby reducing their pairwise sequence divergence; (ii) one genome can recombine with a DNA stretch from an outgroup genome, increasing the pairwise divergence. While several recombination-aware phylogenetic algorithms exist, many of these cannot account for both types of recombination; some algorithms can, but do so inefficiently. Moreover, many of them reconstruct the ancestral recombination graph (ARG) to help infer the genome tree, and require that a substantial portion of each genome has not been affected by recombination, a sometimes unrealistic assumption.

**Methods:**

Here, we propose a Coarse-Graining approach for Phylogenetic reconstruction (CGP), which is recombination-aware but forgoes ARG reconstruction. It accounts for the tendency of a higher effective recombination rate between genomes with a lower phylogenetic distance. It is applicable even if all genomic regions have experienced substantial amounts of recombination, and can be used on both nucleotide and amino acid sequences. CGP considers the local density of substitutions along pairwise genome alignments, fitting a model to the empirical distribution of substitution density to infer the pairwise coalescent time. Given all pairwise coalescent times, CGP reconstructs an ultrametric tree representing vertical inheritance.

**Results:**

Based on simulations, we show that the proposed approach can reconstruct ultrametric trees with accurate topology, branch lengths, and root positioning. Applied to a set of *E. coli* strains, the reconstructed trees are most consistent with gene distributions when inferred from amino acid sequences, a data type that cannot be utilized by many alternative approaches.

**Conclusions:**

The CGP algorithm is more accurate than alternative recombination-aware methods for ultrametric phylogenetic reconstructions.

## Background

The transfer of DNA stretches from one prokaryotic genome to another—also called horizontal gene transfer (HGT) or lateral gene transfer (LGT)—is a major driver of prokaryotic evolution [1]. It is caused by a variety of mechanisms, including transformation, transduction, conjugation, and gene transfer agents [2, 3]. Many prokaryotic genomes encode defense systems against foreign DNA, such as the restriction modification system [4]. A foreign DNA stretch that enters the prokaryotic cell and survives these host defenses may be incorporated into the host genome. If the incoming DNA stretch is highly similar to a stretch on the host genome, homologous recombination may occur, where the incoming DNA stretch homologously recombines with the host stretch and overwrites it [5, 6]. Alternatively, the incoming stretch may be inserted directly into the host genome through non-homologous recombination.

HGT allows the fast spread of genes in prokaryotic pangenomes, and facilitates rapid adaptation to environmental changes. A point in case is the spread of antibiotic resistance genes in pathogenic bacteria via HGT [7]. But recombination is also crucial for the long-term maintenance of prokaryotic populations, as it helps to repair DNA damaged by deleterious mutations, thereby avoiding the mutational meltdown of Muller’s ratchet [8]. In that sense, prokaryotic recombination may fulfill the same function as does sex in eukaryotes. Computational modelling also suggests that recombination may help prokaryotes to purge selfish mobile genetic elements [9].

Recombination can severely affect phylogeny reconstructions. Its effects on genome divergence are complex. Depending on the circumstances, recombination can speed up the divergence of a genome pair or slow it down [6]; its effects may severely affect the accuracy of estimated branch lengths of phylogenetic tree. For example, (i) when a stretch of genome X is replaced by DNA from genome Y, some of the single nucleotide polymorphisms that previously differentiated X and Y will be erased, shortening the apparent evolutionary distance between the two genomes. Conversely, (ii) when X recombines with a DNA stretch of an outgroup genome (a genome that diverged before the split of the X and Y lineages), then it introduces further nucleotide polymorphisms into X, thereby increasing the apparent X-Y distance.

Multilocus sequence typing (MLST) aims to extract sequences of housekeeping genes from prokaryotic genomes, which can then be utilized to resolve evolutionary relationships [10]. However, MLST genes may also experience frequent recombination, and phylogenetic reconstruction without accounting for recombination can compromise the resulting trees [11]. In fact, the frequency with which recombination affects a gene can be of the same order of magnitude as the corresponding mutation rate [5]. Thus, if two lineages recombine with each other, application of conventional phylogenetic algorithms without accounting for recombination will generally lead to an underestimation of the age of their common ancestor [12]. When there are more than two strains, recombination affects the reliability of inference of relative divergence times between the strains and may hence compromise both tree topology and branch length estimation.

There are several popular recombination-aware algorithms, including ClonalFrameML (and its predecessor ClonalFrame) [13, 14], the Bacter package in BEAST2 (which implements the ClonalOrigin model) [15, 16], and Gubbins [17]; there are also non-phylogenetic algorithms that detect recombination, such as BratNextGen and fastGEAR [18, 19]. These recombination-aware algorithms may reconstruct the ARG, which describes the history of transfer and homologous recombination of local genomic stretches across the genomes, to help infer the tree of phylogenetic inheritance of the genomes. While these algorithms can identify genomic stretches with high numbers of substitutions due to recombination with distant strains and thus account for type (ii) recombination effects, many do not take type (i) recombination effects into account; Bacter can account for type (i) effects, but is not computationally efficient for long genome sequences. Some of these algorithms rely on the assumption of low recombination rates, such that a substantial part of a genome remains clonal and has not been affected by recombination. This is unrealistic at least for some bacteria: e.g., for a pair of *E. coli* strains whose DNA sequences have diverged by more than 1.3%, the two share very few stretches larger than a few kb that have not been affected by recombination [5]. Moreover, ARG may only reveal the latest recombination events on a genomic stretch, but its ability to recover the earlier events on the same stretch is limited, since each recombination erases the history of previous recombinations; this uncertainty on earlier recombination events may introduce error in branch length prediction.

In this paper, we propose a novel approach to phylogenetic reconstruction that neither assumes low recombination rates nor relies on ARG reconstruction. Our approach follows a coarse-graining model, which considers the local density of substitutions on a sequence alignment instead of site-specific substitutions [5]. The model describes how different parameters, such as mutation rate, recombination rate, or coalescent time between a pair of genomes, affect the shape of their distribution of substitution density. It accounts for the tendency of a higher level of effective recombinations between genomes with a lower phylogenetic distance: while the model imposes a constant, distance-independent rate of DNA fragments transferring from one genome to another, it also imposes a success rate on the subsequent homologous recombination that is exponentially decaying with increasing sequence divergence between the incoming DNA fragment and the host genome; thus, this effectively renders a declining trend of homologous recombination for genomes with increasing phylogenetic distance. In our algorithm, it fits the empirical distribution of substitution density of genome pairs to the model, which allows the inference of the matrix of coalescent time between the genome pairs and thereafter their ultrametric phylogenetic tree. In short, it forgoes the reconstruction of ARG and infers the branch lengths—coalescent times—from the relative abundance of genomic segments with different number of substitutions, and is also applicable to both nucleotide sequences and amino acid sequences. The source code implementing this model is available at https://github.com/TinPang/coarse-graining-phylogenetics.

## Results

### A coarse-graining approach to phylogenetic reconstruction

**Fig. 1:**
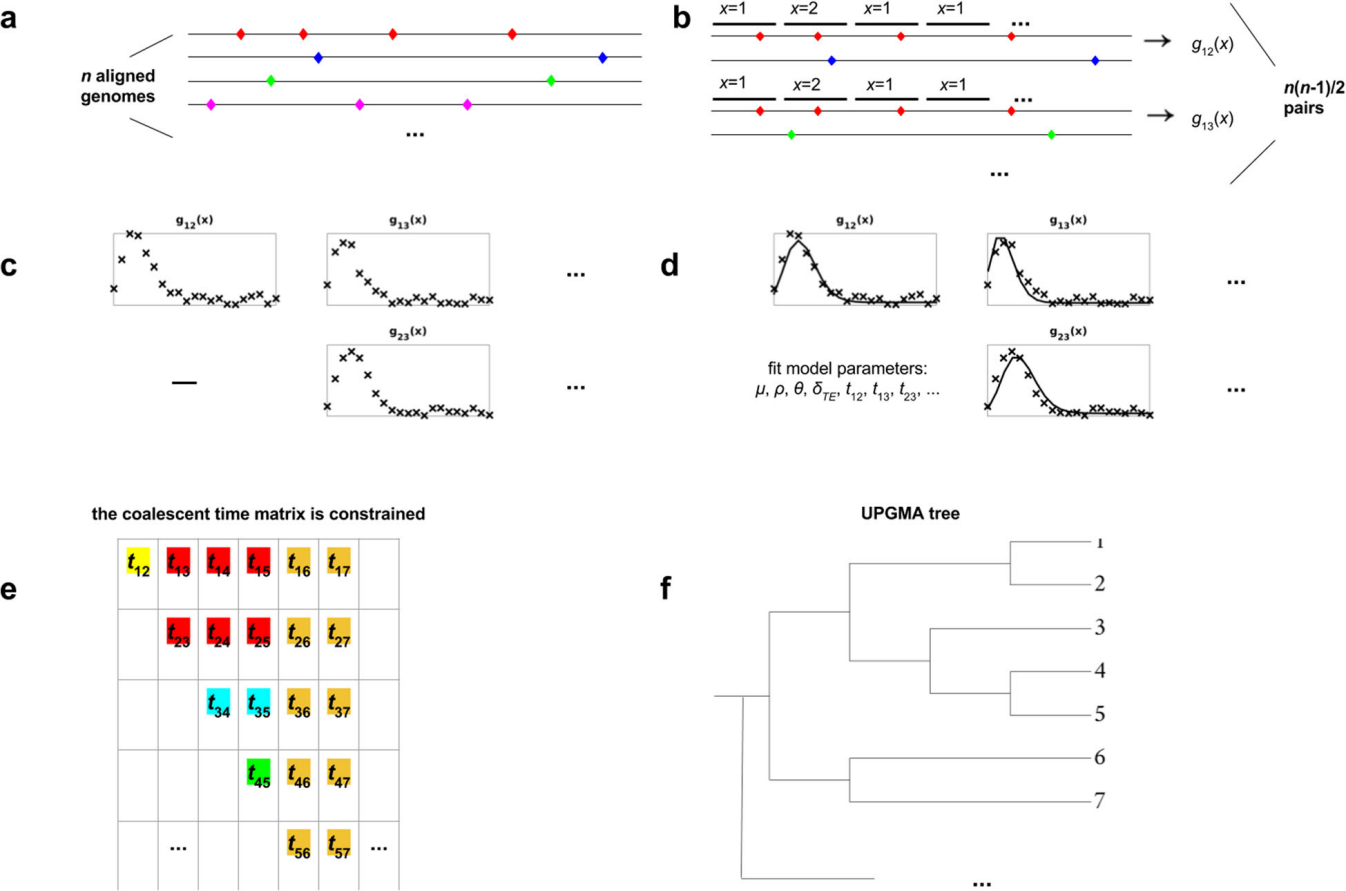
Illustration of the procedure of the proposed CGP algorithm. **a** The algorithm takes *n* aligned sequences as input, which can be nucleotide sequences or amino acid sequences; substitutions on the sequences are represented by coloured markers. **b** Each of the *n*(*n*-1)/2 genome pairs is divided into equal-sized segments, and the pairwise substitutions on each segment is enumerated to obtain the distribution of local SSPs density (denoted as *g*(*x*)). **c** The algorithm aims to infer the distance matrix of the genome sequence pairs from the *n*(*n*-1)/2 SSP distributions. **d** In particular, the algorithm fits the empirical SSP distributions with a model; the input of this model involves a matrix of *n*(*n*-1)/2 coalescent times and other model parameters (mutation rate *μ*, recombination rate *ρ*, average population divergence *θ* and transfer efficiency *δ*_*TE*_). **e** In the fitting process, the *n*(*n*-1)/2 coalescent times are constrained (matrix cells with the same colour have the same value), such that the matrix can be bijectively mapped to a UPGMA tree. **f** the algorithm explores the model parameter space and tree space to obtain the best fit ultrametric tree

**Fig. 2:**
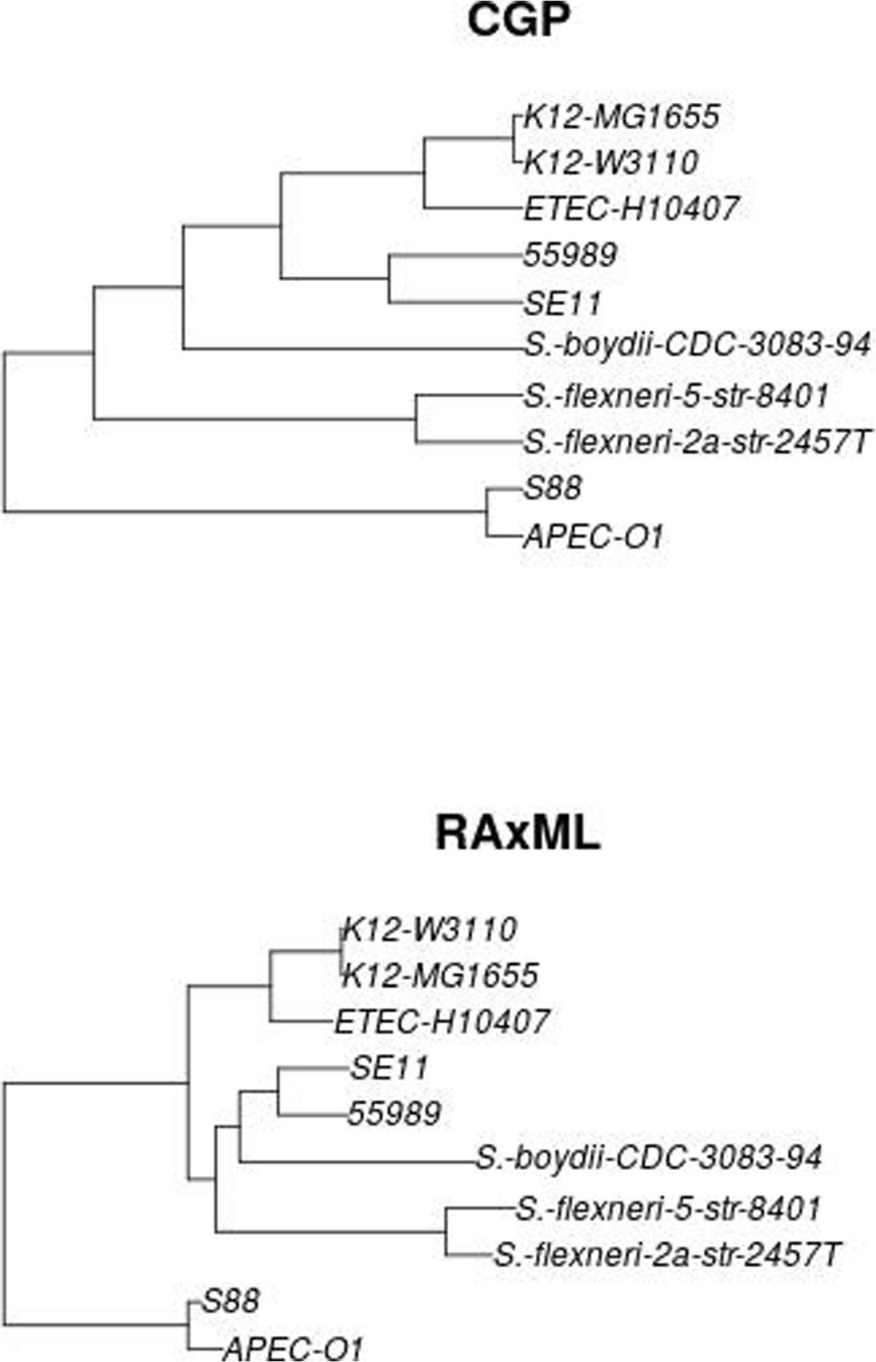
Phylogenetic trees of 10 *E. coli* / *Shigella* strains reconstructed by CGP and RAxML from their amino acid sequences of their core genes. They constitute one of the 100 test-groups in the GLOOME-test. See Supplementary Text in Additional File 5 for details of the GLOOME-test, and Figure S3 for the trees reconstructed by other algorithms

Figure 1 gives a brief illustration on how the proposed CGP algorithm fits the distribution of local single site polymorphisms (SSPs) density of the genome pairs to infer their phylogenetic tree, forgoing the reconstruction of ARG. In short, CGP is based on a mathematical model [5, 6] that quantitatively describes the evolution of genomic sequence divergence; this model is applicable to both nucleotide sequences and amino acid sequences, and does not assume low recombination rate. Recombination can introduce DNA stretches characterized by a high density of substitutions, and the model considers substitution densities defined on the genomic segments. A nucleotide sequence alignment (or a corresponding concatenation of amino acid sequence alignments) is divided into a chain of consecutive, non-overlapping segments, each with *l*_*s*_ sites; for a pair of genomes, we enumerate the SSPs on each segment to obtain the SSP distribution. CGP algorithm takes the SSP distribution of every pair of considered genomes as input. The coalescent time of two genomes can be inferred by fitting CGP's model to the empirical SSP distribution. The ultrametric tree describing the vertical component of inheritance among *n* genomes can be inferred from the coalescent times resulting from the fits to the *n*(*n*-1)/2 empirical SSP distributions, implemented by the score function of Eq. (3) (Methods). We developed the CGP algorithm, which employs Monte Carlo simulation to sample the model+tree space, identifying the tree and parameters that result in the highest score. As an example, Fig. 2 compares the phylogenetic trees reconstructed by CGP and RAxML, and Figure S3 for trees reconstructed by more different algorithms.

### Testing the CGP algorithm on simulated genomes

We performed Fisher-Wright simulations with recombination to generate genome sequences, allowing us to test different phylogenetic reconstruction algorithms. In the simulation, each recombination-attempting DNA stretch starts at a random site of a genome, with equal chance to be either a micro (geometric distribution, mean 100 bp) or a macro stretch (geometric distribution, mean 1 kb). We used three sets of parameters that correspond to prokaryotic populations with r/m = 2, 40, 80 (r/m is the ratio between substitutions contributed by mutations and by recombinations; these three settings are denoted as low, intermediate, and high recombination levels, respectively), and prepared the test groups, each with 4–10 genome sequences. For comparison, r/m values observed in nature range from 0.02 to 63.6 [11].

The most recent common ancestor (MRCA) of a group of random genomes in a simulated population has an average age close to the age of the population root node *t*_root_. We would like to mimic the condition where a single lineage diverges from the rest of the population and forms its own subpopulation, so that the genomes in its subpopulation continue exchanging DNA among themselves and with the rest outside. Hence, when picking genomes in the population to form test groups, we constrained the age of the MRCA of the genomes in a test group, *t*_test-group-root_, to be *t*_test-group-root_≪*t*_root_ (see Supplementary Text in Additional File 5 for the details, and Additional File 4 for the genome sequences in each test-group).

We applied CGP, as well as the previously published methods RaxML [20], ClonalFrameML [14], and Gubbins [17] to the sequences of each test group. The RAxML and Gubbins trees are midpoint-rooted. ClonalFrameML requires an initial tree as input and we used the mid-pointed-rooted RAxML tree. We used segment size *l*_*s*_ = 150 and *l*_*s*_^*cutoff*^ = 100 in CGP. We compared each reconstructed tree with the true tree, measuring their unrooted symmetric distance (SD) [21], as well as their rooted and unrooted branch score distance (BSD) [22] to quantify the accuracy of the reconstructed phylogeny (see Additional File 1 for the these values); the lower the unrooted SD / unrooted BSD / rooted BSD, the more accurate is topology / topology and branch lengths / topology and branch lengths and root positioning, respectively. We normalized the branch lengths of each tree by its total branch length when calculating BSD.

**Fig. 3:**
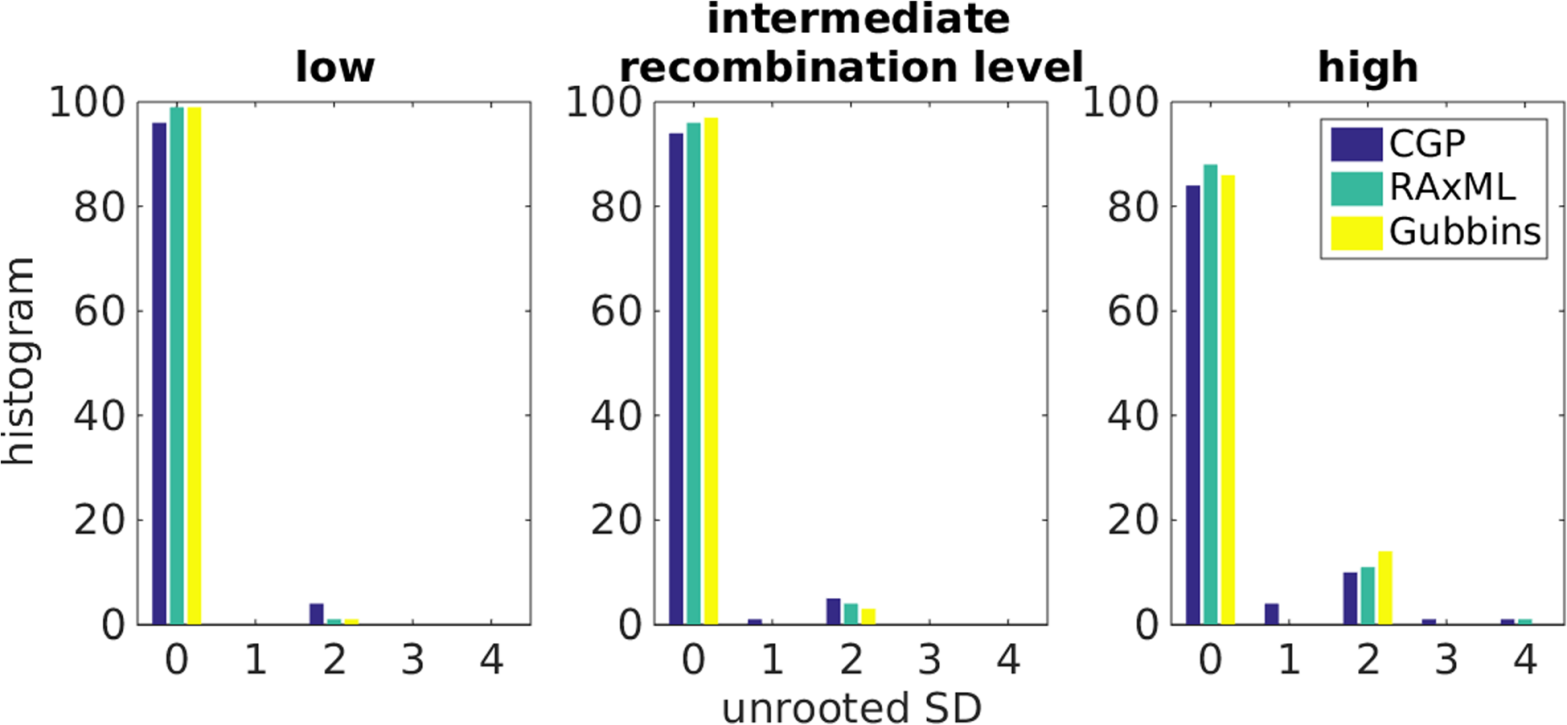
Histograms showing the distributions of the unrooted symmetric distance (SD) between true trees and trees reconstructed by CGP, RaxML, and Gubbins from genome sequences derived from Fisher-Wright simulations with low, intermediate, and high recombination levels. Notice that an unrooted SD value of 0 corresponds to topological consistency between the true tree and the reconstructed tree, which is 96, 94, and 84% at low, intermediate, and high levels of recombination for CGP

CGP can predict the topology of a phylogeny of vertical inheritance as accurately as the other algorithms. We quantified the topological deviation between two trees by unrooted SD, which is defined as the number of phylogenetic clusters that are found in only one of the two; hence, an unrooted SD value of 0 corresponds to correct topological prediction. Figure 3 shows the histogram of unrooted SD values of different algorithms; ClonalFrameML is excluded as it uses the topology of RAxML trees. The rate of correct prediction decreases with increasing level of recombination for all algorithms; at low, intermediate, and high recombination levels, CGP’s correct prediction rate is 96, 94, and 84%, respectively. In addition, the distributions of unrooted SD of CGP are not significantly different from the distributions of RAxML and Gubbins. Two-sided Wilcoxon signed-rank tests (WSRT) at low, intermediate, and high recombination levels resulted in *p* = 0.25, 0.69, 0.54 between CGP and RAxML, and *p* = 0.25, 0.38, 0.92 between CGP and Gubbins.

**Fig. 4:**
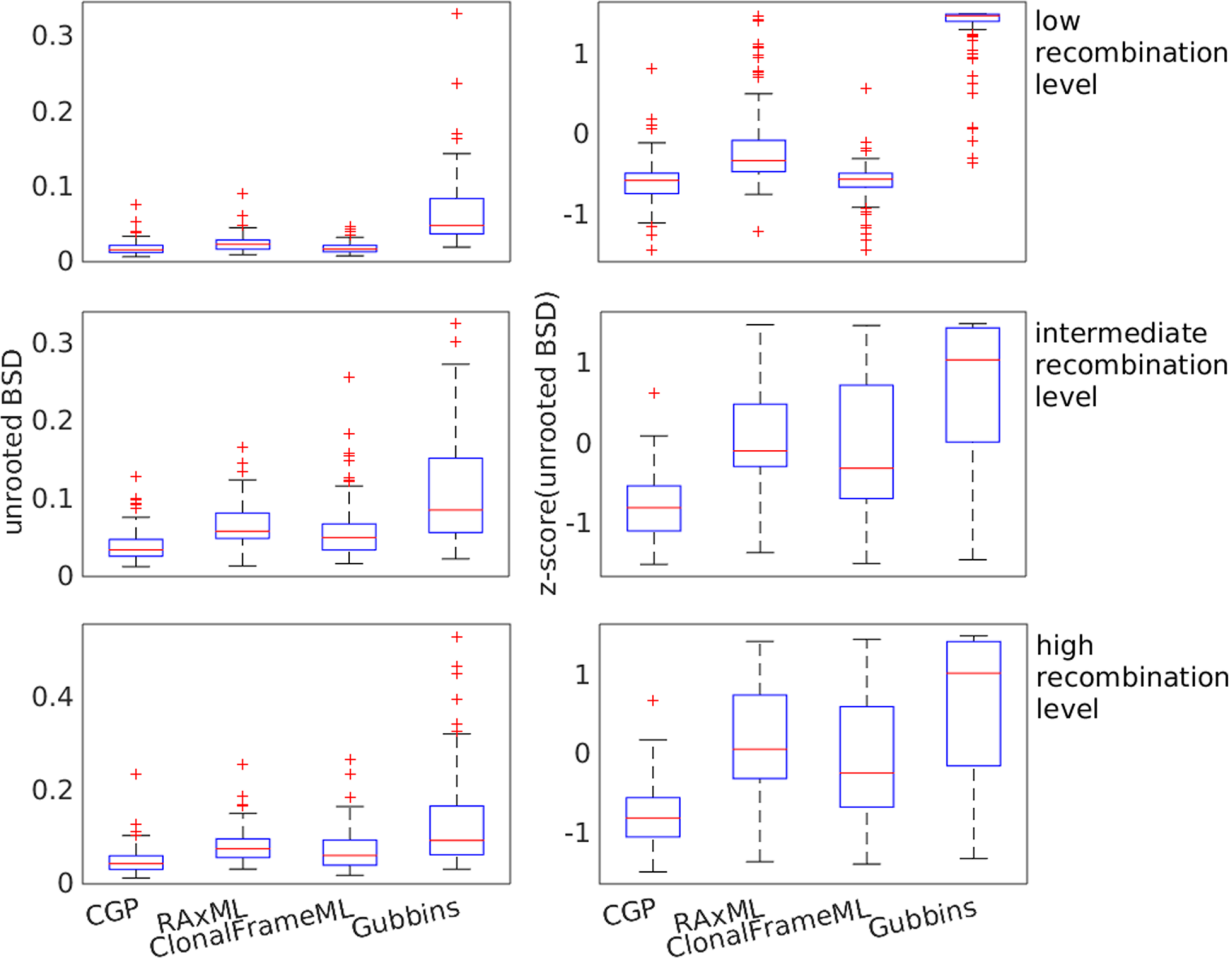
Boxplots showing the distributions of the unrooted branch score distance (BSD) and the distributions of the z-score of unrooted BSD between the true trees and trees reconstructed by CGP, RaxML, and Gubbins from genome sequences from Fisher-Wright simulations with low, intermediate, and high recombination levels

Branch length predictions are more accurate with CGP than with alternative programs at a higher level of recombination. Figure 4 plots the distributions of the unrooted BSD of different algorithms and their z-scores; the unrooted BSD of trees reconstructed by different algorithms on the same test group sequences are pooled together to calculate their z-scores to help data visualization. The distribution of the unrooted BSD of CGP is significantly lower than RAxML and Gubbins (WSRT, *p* < 10^− 10^ at all recombination levels compared to both RAxML and Gubbins). The unrooted BSD of CGP is significantly lower than ClonalFrameML except at low recombination level (WSRT, *p* = 0.76, 2.2 × 10^− 7^, 10^− 7^ at low, intermediate, and high recombination levels, respectively).

**Fig. 5:**
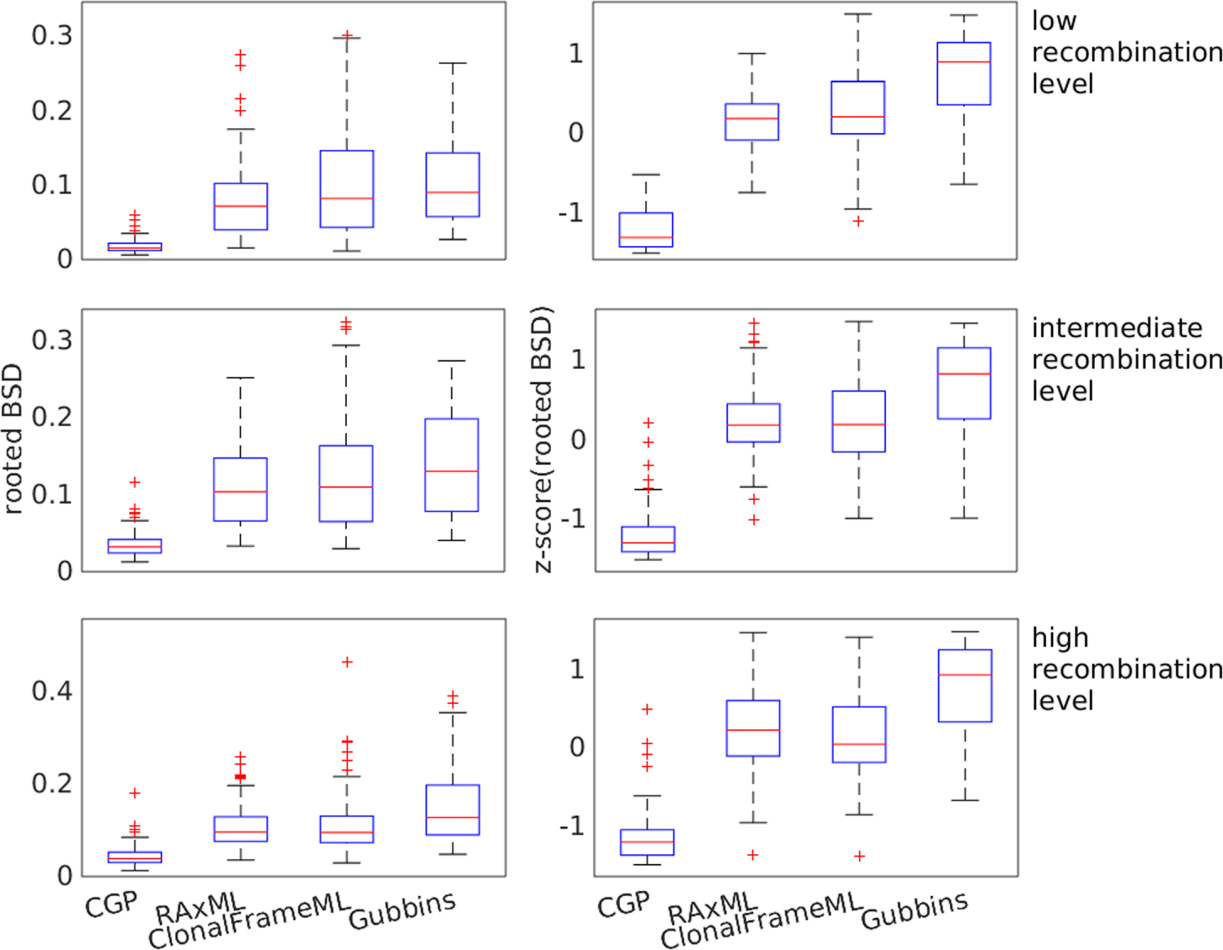
Boxplots showing the distributions of the rooted branch score distance (BSD) and the distributions of the z-score of rooted BSD between the true trees and trees reconstructed by CGP, RaxML, and Gubbins from genome sequences from Fisher-Wright simulations with low, intermediate, and high recombination levels

CGP can perform accurate root positioning. Figure 5 plots the distribution of the rooted BSD and their z-scores; the rooted BSD of trees reconstructed by different algorithms on the same test group sequences are pooled together to calculate their z-scores. The distribution of the rooted BSD of CGP is significantly lower than the other algorithms (WSRT, *p* < 2 × 10^− 17^ at all recombination levels for CGP compared with the other three algorithms).

### Testing the CGP algorithm on real *E. coli* genomes

We tested CGP, RAxML, ClonalFrameML, and Gubbins using *E. coli* and *Shigella* genome sequences (see Supplementary Table S1 in Additional File 5 for their names); we refer to them as *E. coli*, as these two species have intertwined phylogenies. We prepared test groups, each with 10 random strains, where each strain is represented by a nucleotide sequence and an amino acid sequence made from its concatenated core genes (see Additional File 2 for the strains in each test group, and also the 1636 orthologous gene families of core genes; see Additional File 4 for their sequences). We applied CGP, RAxML, ClonalFrameML (with the topology from RAxML trees), and Gubbins on the nucleotide sequences, and CGP and RAxML on amino acid sequences; the RAxML and Gubbins trees are midpoint-rooted. CGP uses segment length *l*_*s*_ = 150 and *l*_*s*_^*cutoff*^ = 100 for nucleotide sequences, and *l*_*s*_ = 50, *l*_*s*_^*cutoff*^ = 50 for amino acid sequences.

We analysed the time for reconstructing each tree to appraise the computational cost of different algorithms (see Supplementary Text in Additional File 5). We found that our CGP is the slowest among the algorithms for reconstructing nucleotide sequences, and also among those for amino acid sequences (Figure S4), as our script is developed as a proof-of-concept and has not been optimized. In general, the run time of our algorithm is slower for nucleotide sequences than amino acid sequences, a consequent of our choice of parameters. Since we used a segment size 150 for nucleotide sequences and 50 for amino acid sequences, it involves the operations of the larger 150 × 150 matrices for nucleotide sequences, which is computationally more demanding compared with the smaller 50 × 50 matrices for amino acid sequences. Further, while the divergence between amino acid sequences is lower than nucleotide sequences, we assumed, for simplicity, the same mutation rate for both types of sequences; hence, it takes more time steps for the algorithm to explore the solution space when using nucleotide sequences, and thus it has a higher computational cost.

**Fig. 6:**
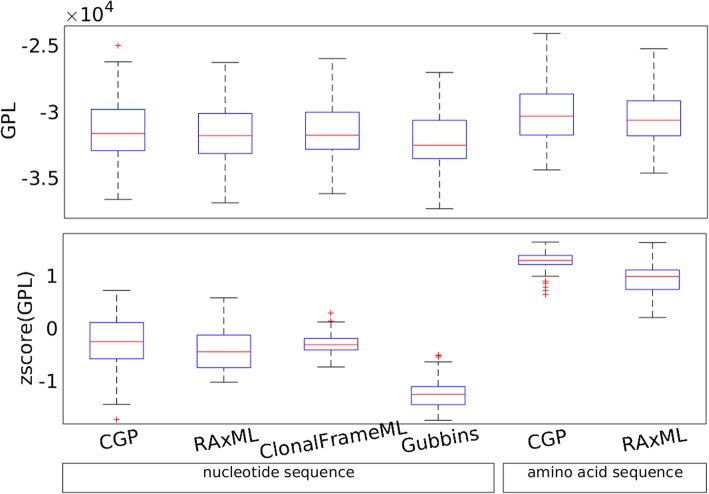
Boxplots showing the distributions of the GLOOME posterior likelihood (GPL) and the distributions of the z-scores of GPL. The trees were reconstructed from observed *E. coli* genome sequences, applying CGP, RAxML, ClonalFrameML, and Gubbins to nucleotide sequences, and CGP and RAxML to amino acid sequences

To assess the accuracy of the phylogenetic trees reconstructed by the different algorithms, we compared the reconstructed trees with the phylogenetic signal inferred from the distribution of orthologous gene families in different genomes. We applied the GLOOME algorithm [23], which considers the interior nodes of the tree as ancestral strains and reconstructs their gene distribution; it takes a tree and the presence-and-absence of genes across the extant strains as input, and performs a reconstruction of presence-and-absence of genes in the ancestral strains based on the GLOOME posterior likelihood (GPL). We used GPL as a score to quantify the accuracy of the tree fed into GLOOME; the more consistent the phylogenetic signal from the gene distributions with a given tree, the higher the GPL (see Additional File 2 for the GPL values of the reconstructed trees). Figure 6 plots the distribution of the GPLs and the corresponding z-scores; the GPLs of trees of the same test groups reconstructed by different methods are pooled together to calculate the z-scores. Trees reconstructed from amino acid sequences have a higher GPL than trees calculated from nucleotide sequences; moreover, CGP trees based on amino acid sequences are more accurate than trees calculated using RAxML (*p* < 4 × 10^− 14^ when comparing CGP on amino acid sequences with any other algorithm; other recombination-aware algorithms are not applicable to amino acid sequences). Considering only trees reconstructed from nucleotide sequences, the CGP trees generally have higher GPL than RAxML, ClonalFrameML, and Gubbins trees (WSRT, *p* = 5.2 × 10^− 4^, 0.049, 1.4 × 10^− 15^, respectively).

Alternatively, we also used the consistency of a phylogenetic algorithm as a proxy indicator of its accuracy. Within a test-group that comprises 10 real genomes, we randomly assigned their core genes to either set A or set B, making a super-gene sequence A and a super-gene sequence B for each genome; tree A and tree B of the group are then reconstructed from the two sets of super-gene sequences, using the same phylogenetic algorithm. We then calculated the unrooted SD / unrooted BSD / rooted BSD between the tree pair of 100 test-groups to quantify the (in)consistency of different algorithms (see Additional File 3 for these scores; see Supplementary Text in Additional File 5 for details); these tree-distance measures are pooled together to calculate their z-score (Figure S5). The unrooted SD between tree pairs reconstructed by CGP applied to nucleotide sequences is lower than RAxML and Gubbins applied to nucleotide sequences, and also RAxML applied to amino acid sequences (WSRT, *p* = 0.0051, 0.0012, 1.2 × 10^− 5^, respectively), but not CGP applied to amino acid sequences (WSRT, *p* = 0.12); CGP applied to amino acid sequences is lower than Gubbins applied to nucleotide sequences and RAxML applied to amino acid sequences (WSRT, *p* = 0.0343, 0.0036, respectively), but not RAxML applied to nucleotide sequences (WSRT, *p* = 0.2273); hence, CGP applied to nucleotide sequences, ignoring the branch length of the trees, has the highest topological consistency. The unrooted BSD for CGP applied to nucleotide sequences is higher than RAxML, ClonalFrameML, and Gubbins applied to nucleotide sequences (WSRT, *p* = 0.015, 0.00014, 0.0032, respectively), as high as CGP applied to amino acid sequences (WSRT, *p* = 0.36), and lower than RAxML applied to amino acid sequences (WSRT, *p* = 0.0183); CGP applied to amino acid sequences is higher than RAxML, ClonalFrameML, and Gubbins applied to nucleotide sequences (WSRT, *p* = 0.0015, 8.6 × 10^− 6^, 3.5 × 10^− 5^, respectively), and lower than RAxML applied to amino acid sequences (WSRT, *p* = 3.0 × 10^− 8^); thus, CGP at best does not outperform other algorithms, except for RAxML applied to amino acid sequences, in terms of topological and branch length consistency. The rooted BSD for CGP applied to nucleotide sequences is as high as RAxML, ClonalFrameML, and Gubbins applied to nucleotide sequences, and also CGP applied to amino acid sequences (WSRT, *p* = 0.63, 0.17, 0.24, 0.70, respectively), but lower than RAxML applied to amino acid sequences (WSRT, *p* = 1.3 × 10^− 5^); moreover, CGP applied to amino acid sequences is higher than ClonalFrameML and Gubbins (WRST, *p* = 0.019, 0.0011, respectively), as high as RAxML applied to nucleotide sequences (WSRT, *p* = 0.12), and lower than RAxML applied to amino acid sequences (WSRT, *p* = 1.9 × 10^− 10^); therefore, CGP is no better than other algorithms, except for RAxML applied to amino acid sequences, in terms of topological, branch length and root-positioning consistency. Some of the findings in this analysis contradict the observations in previous analyses. For example, trees reconstructed from amino acid sequences tend to have a higher GPL—a measure of accuracy—than those reconstructed from nucleotide sequences, whereas here we observed that tree pairs reconstructed from amino acid sequences tend to have a lower consistency (higher unrooted SD / unrooted BSD / rooted BSD) than those reconstructed from nucleotide sequences. When comparing the reconstructed tree with the real tree on simulated sequences, CGP has a lower unrooted and rooted BSD, but not a lower unrooted SD, than other algorithms; however, the opposite trend is observed in this analysis. Therefore, we concluded that this consistency test does not reflect the accuracy of algorithms.

Furthermore, we investigated how the UPGMA assumption perturbs coalescent time inference. For a phylogenetic reconstruction performed by CGP, we obtained its optimal model parameters *μ*, *ρ*, *θ*, and *δ*_*TE*_; we simulated the theoretical model based on these optimal parameters, and fitted the model to the empirical pairwise SSP distributions to directly infer the coalescent time, *t*_direct_, of different sequence pairs. Given the coalescent time inferred from the optimal tree reconstructed by CGP, *t*_tree_, we calculated the deviation between the two coalescent times, Δ, defined as *Δ* = (*t*_*direct*_ − *t*_*tree*_)/(*t*_*direct*_ + *t*_*tree*_), to quantify the perturbation caused by UPGMA assumption (see Supplementary Text in Additional File 5 for details). The distribution of Δ for phylogenetic reconstructions based on nucleotide sequences, and also on amino acid sequences, are mostly confined between − 0.1 and 0.1 (Figure S6), roughly indicating a less-than-10% perturbation on the inferred branch length.

## Discussion

We introduced the CGP algorithm, which infers phylogenetic trees based on the estimated pairwise coalescent times of genomes. We conducted extensive analyses to compare the accuracy of the CGP algorithm with other state-of-the-art algorithms to demonstrate its ability to reliably predict the topology, branch lengths, and root positioning of phylogenetic trees. CGP's model does not rely on the assumption of low recombination rates; this allows the model to predict branch lengths accurately even if the vast majority of the considered genome segments have experienced recombination.

Analyses performed on the real *E. coli* genome sequences showed that trees reconstructed from core genome amino acid sequences are more accurate, i.e., more consistent with the signal inferred from the distribution of genes in the extant genomes, than trees calculated from nucleotide sequences. Amino acid sequences of core genes tend to evolve more slowly than the corresponding DNA sequences, as these genes show dN/dS values < 1 [24], and accordingly, the divergence of a pair of amino acid sequences is lower than that of their nucleotide sequences counterparts (see Additional File 4 for nucleotide sequence and amino acid sequence divergence between *E. coli* genome pairs). Thus, amino acid sequences may be more “clonal” than nucleotide sequences and thus may provide more accurate phylogenetic signals.

The major source of error of the CGP algorithm comes from the mismatch between the genomic segments as basic unit of the algorithm and the genomic stretches affected by homologous recombination, as the algorithm does not try to match the boundary of the segments to the boundary of the actual recombination stretches. This mismatch gives rise to segments that lie on the boundary and cover multiple recombination stretches, which subsequently reduces the accuracy of the predictions of the algorithm. Hence, a possible direction for further development is to find out the criteria to fine tune the segment size *l*_*s*_ so as to minimize these boundary-overlapping segments; alternatively, we can improve the theoretical model so that the segments do not have to be equal-sized and we can match the segments to the recombination stretches.

The computational demand of the CGP algorithm is independent of sequence length, as CGP considers only SSP distributions that are represented by a vector of 1 + *l*_*s*_^*cutoff*^ elements in the computer code. Calculation of the CGP score (Eq. (3)) involves multiplication of (1 + *l*_*s*_^*cutoff*^) × (1 + *l*_*s*_^*cutoff*^) matrices; thus, the computational time scales as *O*((*l*_*s*_^*cutoff*^)^*k*^), where *k* ≤3 depends on the algorithm that carries out the matrix operations. When reconstructing a tree of *n* genomes, the score calculation involves the summation over *n*(*n*-1)/2 pairs, making it scale as *O*(*n*^2^). The segment size *l*_*s*_ affects the efficiency and accuracy of the algorithm. While a smaller *l*_*s*_ leads to lower accuracy, increasing *l*_*s*_ leads to higher computational demand; a large *l*_*s*_ combined with a small *l*_*s*_^*cutoff*^ can also reduce the accuracy. Hence, one needs to set *l*_*s*_ and *l*_*s*_^*cutoff*^ carefully to balance the need for speed and accuracy.

The current implementation of the CGP algorithm is very simple and basic; it should be considered a proof of concept. While it makes use of Monte Carlo simulation to sample the tree+parameter space, a hill-climbing method may be more efficient. Other possible improvements involve better local search moves in the ultrametric tree space; one might even drop the stringent ultrametricity constraint, and replace it with a more flexible matrix-tree mapping method that allows a more efficient search in the tree space. The mutation matrix in the current model can be improved to include back mutations and a more complex mutation model. We leave these possible improvements to future studies.

## Conclusions

Homologous recombination allows a foreign DNA segment to overwrite a synonymous segment of a bacterial genome, erasing the history of previous nucleotide substitutions and posing a great challenge to the reconstruction of vertical inheritance of the bacterial genomes. Here we propose the CGP algorithm, which is recombination-aware and applicable to both nucleotide and amino acid sequences, to reconstruct ultrametric phylogenetic trees. Unlike most phylogenetic algorithms, which consider individual SSPs on genome sequences, CGP implements an innovative approach that considers the density of SSPs across different local regions of the sequences. By fitting the empirical distributions of SSP density to a theoretical model, CGP infers the coalescent time of different pairs of genome sequences, and thus their ultrametric tree. Analyses in this study compare the CGP algorithm with alternative recombination-aware phylogenetic algorithms, and show that for genomes with frequent recombination CGP can more accurately reconstruct their phylogenetic trees, not least branch length prediction and root positioning, than alternative algorithms.

## Methods

### Overview of the CGP algorithm that reconstructs ultrametric phylogenetic tree

The proposed CGP algorithm takes *n* aligned genome sequences as input (Fig. 1a), which can be either nucleotide sequences or amino acid sequences. For each pair of sequences, it divides them into *L*_*seg*_ equal-sized segments, each segment has *l*_*s*_ sites, and enumerates the SSPs—sites with substitution—on each segment to obtain the distribution of local SSP density of the genome sequence pair (Fig. 1b). This algorithm considers segments instead of nucleotide / amino-acid sites as the basic unit of a genome, because the local SSP density can be defined conveniently on segments; an SSP can be a single nucleotide polymorphism (SNP) on a nucleotide sequence, or a single amino acid polymorphism (SAP) on an amino acid sequence. It then fits the empirical SSP distribution of all pairs to a model to infer the matrix of coalescent time of the genome pairs (Fig. 1d). While searching for the best fit model parameters and pairwise coalescent times, it constrains these *n*(*n*-1)/2 coalescent times (Fig. 1e), so that the matrix can be bijectively mapped to a UPGMA tree that describes the phylogenetic inheritance of the *n* genome sequences (Fig. 1f).

### Model describing the evolution of the SSP distribution of a pair of genomes in a fisher-Wright population

CGP's model, which is used to fit the empirical distribution of local SSP (Fig. 1d), is based on a Fisher-Wright haploid population with non-overlapping generations, constant population size, and homologous recombination [25, 26]. In this framework, a node in one generation inherits the genome of a random node in the previous generation, followed by mutation and homologous recombination. A genome sequence is divided into *L*_*seg*_ consecutive and non-overlapping segments, where every segment has length *l*_*s*_ (i.e., consists of *l*_*s*_ sites). The rate of mutation is *μ* per site per generation. The rate for a site to be covered by a foreign DNA stretch attempting to recombine with the host genome is *ρ* per generation; the rate for a segment to be covered by a recombination-attempting DNA stretch is also approximately *ρ*, assuming that the segment is much shorter than the DNA stretch. Here, *ρ* = *ρ*_*ini*_*L*, where *ρ*_*ini*_ is the probability for a recombination-attempting foreign DNA stretch to start at any given site, and *L* is the average length of the foreign DNA stretch. When a recombination attempt happens on a segment, it either succeeds and the foreign DNA replaces the host DNA at the segment, or it fails. The success rate of an attempt is approximately exp.(−*δ*/*δ*_TE_), where *δ* is the divergence between the incoming DNA and the host DNA, and *δ*_TE_ is the transfer efficiency, a constant that governs the success rate [5]. The average site divergence in the population is denoted as *θ*, with *θ* = 2μN_e_ and population size *N*_*e*_.

CGP’s model [5, 6] considers the evolution of an SSP distribution between a pair of genomes, X and Y. As the alignment of genome X and Y is divided into *L*_*seg*_ consecutive and non-overlapping segments with *l*_*s*_ sites, let *f*(*x*|*t*) be the distribution of segment divergence, where *x* = 0, 1, …, *l*_*s*_ represents the number of SSPs on a segment of the XY alignment, *t* ≥ 0 is the (continuous) XY coalescent time, and *f*(*x*|*t*) is normalized to unity (summing over *x)*. To save computational resources, we assume an upper bound *l*_*s*_^*cutoff*^ ≤ *l*_*s*_ to *x*. At *t* = 0, the MRCA of XY splits into two lineages; initially, the two have identical genomes, and thus *f*(*x*|0) = δ_*x*,0_ (where δ_*x*,0_ is the Kronecker delta, i.e., *f*(*x*|0) is non-zero only at *x* = 0). At *t* > 0, mutations and recombinations occur, and the evolution of *f*(*x*|*t*) is described by the following equation:

1 $$ \frac{df\left(x|t\right)}{dt}=2{l}_s\mu {\sum}_{y=0}^{{l_s}^{cutoff}}\left(M\left(x|y\right)-I\left(x|y\right)\right)f\left(y|t\right)+2\rho {\sum}_{y=0}^{{l_s}^{cutoff}}\left(P\left(x|y,\theta, {\delta}_{TE},{l}_s\right)-I\left(x|y\right)\right)f\left(y|t\right) $$

The first term of Eq. (1) accounts for mutations on a segment—M(*x*|*y*) models a mutation event, where a segment in the pair XY with *y* SSPs increases to *x* = *y* + 1 SSPs during a mutation (i.e.*,* M(*x*|*y*) = 0 for *x* ≠ *y* + 1); I(*x*|*y*) is the identity matrix. For simplicity, we ignore back mutations.

The second term accounts for recombination—P(*x*|*y*,*θ,δ*_*TE*_,*l*_*s*_) models a recombination event (see Eq. (S4) of *Dixit* et al. [5] or Eq. (3) of *Dixit* et al. [6] for a detailed derivation). Since a segment can recombine with its counterpart on another genome, the model assumes that each segment of a genome, along with its counterparts in different genomes of the population, have their own phylogeny that is independent of the genomes’ phylogeny, and the segment population structure is approximated by the coalescent model. For an attempted recombination between Y and an external donor D, we can use the coalescent model to calculate the probability distribution for the segment divergence *δ* between D and X, and obtain *x* from *x* = *l*_*s*_*δ*. As mutation and recombination can equally occur on either X or Y, there is a factor 2 attached to both terms. See Supplementary Text in Additional File 5 for the exact form of P(*x*|*y*,*θ,δ*_*TE*_,*l*_*s*_). We solved Eq. (1) with boundary condition *f*(*x*|0) = δ_*x*,0_ to obtain the theoretical SSP distribution *f*(*x*|*t*) at different coalescent times *t*.

We fit the theoretical distribution predicted from CGP's model to an empirical SSP distribution to infer the coalescent time of its genome pair. Let us consider an alignment for a genome pair XY that is divided into *L*_*seg*_ segments, with empirical SSP distribution *g*_XY_(*x*) following the normalization condition:

$$ {\sum}_{x=0}^{{l_s}^{cutoff}}{g}_{XY}(x)={L}_{seg} $$

Let us denote the theoretical distribution as *f*_*μ,ρ,θ,δTE*_(*x*|*t*), which is normalized to unity. The probability to observe the empirical distribution *g*_XY_(*x*) given the theoretical distribution *f*_*μ,ρ,θ,δTE*_(*x*|*t*) is

2 $$ {\prod}_x{\left[{f}_{\mu, \rho, \theta, {\delta}_{TE}}\left(x|t\right)\right]}^{g_{XY}(x)} $$

If we take the logarithm of this expression, it becomes the (negative) cross entropy between *g*(*x*) and *f*_*μ,ρ,θ,δTE*_(*x*|*t*) [27, 28]. The higher their similarity, the higher is this negative cross entropy; it attains its maximum when *f*_*μ,ρ,θ,δTE*_(*x*|*t*) is equal to *g*(*x*).

Suppose that we have *n* genomes (X_*i*_, *i* = 1,...,*n*), where their phylogeny is described by an ultrametric tree *T*; the *n*(*n*-1)/2 pairwise SSP distributions have evolved according to the model with parameters *μ, ρ, θ, δ*_*TE*_. Let *t*_*T*_ (X_*a*_,X_*b*_) be the coalescent time of X_*a*_ and X_*b*_ in the tree *T*. We use a score function, *S*(X_1_,X_2_, …,X_*n*_|*μ*, *ρ*, *θ*, *δ*_TE_,*T*), which is defined as the logarithm of the probability to observe the *n*(*n*-1)/2 empirical SSP distributions given the model and the tree, to quantify the model fit to the empirical SSP distributions. This score is the summation of the *n*(*n*-1)/2 negative mutual entropy terms:

3 $$ S\left({X}_1,\dots {X}_n|\mu, \rho, \theta, {\delta}_{TE},T\right)={\sum}_{all\ \left({X}_a,{X}_b\right)\  pairs}\mathit{\log}\left\{{\prod}_x{\left[{f}_{\mu, \rho, \theta, {\delta}_{TE}}\left(x|{t}_T\left({X}_a,{X}_b\right)\right)\right]}^{g_{X_a{X}_b}(x)}\right\} $$

Since the *n*(*n*-1)/2 SSP distributions are not completely independent of each other, Eq. (3) is not exactly a probability and so we call it a score. We developed an algorithm that samples the tree+model space and searches for the configuration with the maximum score using Monte Carlo simulation with annealing and Metropolis acceptance (See Supplementary Text in Additional File 5 for details).

## Supplementary information

**Additional file 1.** Analyses of the simulated genomes: the symmetric distance (SD) and branch score distance (BSD) between the reconstructed trees and the true trees.

**Additional file 2 **Analyses of the real genomes in the GLOOME-test: b-number of the *E. coli* core genes used to make the ‘super-gene’ sequences, strains in each test-group, and also the GLOOME posterior likelihood (GPL) of the reconstructed trees.

**Additional file 3.** Analyses of the real genomes in the tree-pair-test: the unrooted symmetric distance, the unrooted branch score distance, and the rooted branch score distance between the pair of reconstructed trees in each test group.

**Additional file 4 **Zipped folder containing the sequences and trees in the analyses: sequences of the simulated genomes in different test-groups, their true trees and also the phylogenetic trees reconstructed by different algorithms; sequences of the *E. coli* genomes, their trees reconstructed by different algorithms for the GLOOME-test, and also their time measurements; sequences of the *E. coli* genomes, the list of strains and genes in each test group, and their trees reconstructed by different algorithms for the tree-pair-test; genes to orthologous gene families map provided by ProteinORTHO.

**Additional file 5.** Supplementary PDF: Supplementary Text, Supplementary Figures and Supplementary Tables.

## Data Availability

All data generated or analysed during this study are included in this published article, its additional files, and GitHub repository (https://github.com/TinPang/coarse-graining-phylogenetics).

## Abbreviations

ARG: Ancestral Recombination Graph
BEAST: Bayesian Evolutionary Analysis by Sampling Trees
BSD: Branch Score Distance
CGP: Coarse-Graining approach for Phylogenetic reconstruction
DNA: DeoxyriboNucleic Acid
GLOOME: Gain LOss Mapping Engine
GPL: GLOOME Posterior Likelihood
HGT: Horizontal Gene Transfer
LGT: Lateral Gene Transfer
MAFFT: Multiple Alignment using Fast Fourier Transform
MLST: MultiLocus Sequence Typing
MRCA: Most Recent Common Ancestor
RAxML: Randomized Axelerated Maximum Likelihood
SAP: Single Amino-acid Polymorphism
SD: Symmetric Distance
SNP: Single Nucleotide Polymorphism
SSP: Single Site Polymorphism
UPGMA: Unweighted Pair Group Method with Arithmetic mean
WRST: Wilcoxon Rank Sum Test

## References

[1] Pál C, Papp B, Lercher MJ (2005). Adaptive evolution of bacterial metabolic networks by horizontal gene transfer. Nat Genet.

[2] Ochman H, Lawrence JG, Groisman EA (2000). Lateral gene transfer and the nature of bacterial innovation. Nature..

[3] Lang AS, Zhaxybayeva O, Beatty JT (2012). Gene transfer agents: phage-like elements of genetic exchange. Nat Rev Microbiol.

[4] Wilson GG, Murray NE (1991). Restriction and modification systems. Annu Rev Genet.

[5] Dixit PD, Pang TY, Studier FW, Maslov S (2015). Recombinant transfer in the basic genome of Escherichia coli. Proc Natl Acad Sci U S A.

[6] Dixit PD, Pang TY, Maslov S. Recombination-driven genome evolution and stability of bacterial species. bioRxiv. 2016:067942. 10.1534/genetics.117.300061 PMC5586378 28751420

[7] Huddleston JR (2014). Horizontal gene transfer in the human gastrointestinal tract: potential spread of antibiotic resistance genes. Infect Drug Resist.

[8] Takeuchi N, Kaneko K, Koonin E (2014). Horizontal Gene Transfer Can Rescue Prokaryotes from Muller’s Ratchet: Benefit of DNA from Dead Cells and Population Subdivision. G3 GenesGenomesGenetics.

[9] Croucher NJ, Mostowy R, Wymant C, Turner P, Bentley SD, Fraser C (2016). Horizontal DNA transfer mechanisms of Bacteria as weapons of Intragenomic conflict. PLoS Biol.

[10] Spratt BG (1999). Multilocus sequence typing: molecular typing of bacterial pathogens in an era of rapid DNA sequencing and the internet. Curr Opin Microbiol.

[11] Vos M, Didelot X (2009). A comparison of homologous recombination rates in bacteria and archaea. ISME J.

[12] Schierup MH, Hein J (2000). Consequences of recombination on traditional phylogenetic analysis. Genetics..

[13] Didelot X, Falush D (2007). Inference of bacterial microevolution using Multilocus sequence data. Genetics..

[14] Didelot X, Wilson DJ (2015). ClonalFrameML: efficient inference of recombination in whole bacterial genomes. PLoS Comput Biol.

[15] Didelot X, Lawson D, Darling A, Falush D (2010). Inference of homologous recombination in Bacteria using whole-genome sequences. Genetics..

[16] Vaughan TG, Welch D, Drummond AJ, Biggs PJ, George T, French NP (2017). Inferring ancestral recombination graphs from bacterial genomic data. Genetics..

[17] Croucher NJ, Page AJ, Connor TR, Delaney AJ, Keane JA, Bentley SD (2015). Rapid phylogenetic analysis of large samples of recombinant bacterial whole genome sequences using Gubbins. Nucleic Acids Res.

[18] Marttinen P, Hanage WP, Croucher NJ, Connor TR, Harris SR, Bentley SD (2012). Detection of recombination events in bacterial genomes from large population samples. Nucleic Acids Res.

[19] Mostowy R, Croucher NJ, Andam CP, Corander J, Hanage WP, Marttinen P. Efficient inference of recent and ancestral recombination within bacterial populations. bioRxiv. 2017:059642. 10.1093/molbev/msx066 PMC5400400 28199698

[20] Stamatakis A. RAxML Version 8: A tool for Phylogenetic Analysis and Post-Analysis of Large Phylogenies. Bioinformatics. 2014:btu033. 10.1093/bioinformatics/btu033 PMC3998144 24451623

[21] Robinson DF, Foulds LR (1981). Comparison of phylogenetic trees. Math Biosci.

[22] Kuhner MK, Felsenstein J (1994). A simulation comparison of phylogeny algorithms under equal and unequal evolutionary rates. Mol Biol Evol.

[23] Cohen O, Ashkenazy H, Belinky F, Huchon D, Pupko T (2010). GLOOME: gain loss mapping engine. Bioinformatics..

[24] Lapierre M, Blin C, Lambert A, Achaz G, Rocha EPC (2016). The impact of selection, gene conversion, and biased sampling on the assessment of microbial demography. Mol Biol Evol.

[25] Kingman JFC (2000). Origins of the coalescent: 1974-1982. Genetics..

[26] Fraser C, Hanage WP, Spratt BG (2007). Recombination and the nature of bacterial speciation. Science..

[27] Rubinstein RY, Kroese DP (2004). The Cross-Entropy Method: A Unified Approach to Combinatorial Optimization, Monte-Carlo Simulation and Machine Learning. Springer Science & Business Media.

[28] de Boer P-T, Kroese DP, Mannor S, Rubinstein RY (2005). A tutorial on the cross-entropy method. Ann Oper Res.

