# Supplementary material for: A coarse-graining, ultrametric approach to resolve the phylogeny of prokaryotic strains with frequent homologous recombination
Source: BMC Evol Biol. 2020 May 7;20:52. doi: 10.1186/s12862-020-01616-5 (PMC7204016; doi:10.1186/s12862-020-01616-5)
Supplement: Supplementary file 5 — Additional file 5. Supplementary PDF: Supplementary Text, Supplementary Figures and Supplementary Tables. [file 12862_2020_1616_MOESM5_ESM.pdf]

SUPPLEMENTARY TEXT

Theoretical model to describe the evolution of SSP distribution

The model behind the CGP algorithm is derived from the theoretical work introduced in *Dixit et al.* (Dixit et al. 2015, 2017), which describes the evolution of the distribution of substitution density between a pair of genomes. Let us divide the sequence alignment of a pair of genomes, X and Y, into L_{seg} consecutive and non-overlapping segments, where each segment has l_s sites. A sequence here can be a nucleotide sequence or an amino acid sequence, and a polymorphic site on the alignment is called single site polymorphism (SSP); this corresponds to a single nucleotide polymorphism (SNP) of a nucleotide sequence, or a single amino-acid polymorphism (SAP) of an amino-acid sequence. Let $f(x|t)$ be the distribution of SSPs on the segments, where x=0, 1, …, l_s represents the number of SSPs on a segment of the XY alignment, t≥0 be the XY coalescent time. To save computational resource, let us assume an upper bound $l_s^{cutoff} \leq l_s$ to x, where segments with $x > l_s^{cutoff}$ SSPs are simplified to have l_s^{cutoff} SSPs. $f(x|t)$ is normalized to unity in the following way:

$$\sum_{x=0}^{l_s^{cutoff}} f(x|t) = 1$$

At t=0, the most recent common ancestor (MRCA) of XY splits into two lineages; the two have identical genomes, and thus $f(x|0)=\delta_{x,0}$ (Kronecker delta, *i.e.*, this is non-zero only at x=0). When t>0, mutations and recombinations occur, and the evolution of $f(x|t)$ is described by the following equation (Eq. (1) in the main text):

$$\frac{df(x|t)}{dt} = 2l_s\mu \sum_{y=0}^{l_s^{cutoff}} \left(M(x|y) - I(x|y)\right) f(y|t) + 2\rho \sum_{y=0}^{l_s^{cutoff}} \left(P(x|y,\theta,\delta_{TE},l_s) - I(x|y)\right) f(y|t)$$

The first term of this equation accounts for mutation, and the second term accounts for recombination; there is a factor 2 because mutation and recombination can equally occur on either X or Y. μ is the mutation rate per site, and $l_s\mu$ is the mutation rate per segment; $I(x|y)$ is the identity matrix; $M(x|y)=\delta_{x,y+1}+(\delta_{x,lscutoff}\times\delta_{y,lscutoff})$ is the mutation matrix (*i.e.,* $M(x|y)=0$ for $x \neq y+1$), as a segment with y SSPs jumps to $x=y+1$ SSPs after a mutation. For simplicity, it does not consider back mutation. ρ is the rate for a segment to be covered by a recombination stretch. The value of μ or ρ changes if time is rescaled, but not ρ/μ. $P(x|y,\theta,\delta_{TE},l_s)$ is the recombination matrix, which is the probability for a segment to change its number of SSPs from y to x during a recombination. $P(x|y,\theta,\delta_{TE},l_s)$ corresponds to Eq. (S4) in *Dixit et al.* (Dixit et al. 2015) and Eq. (3) in *Dixit et al.* (Dixit et al. 2017); these literatures describe the detailed derivation of $P(x|y,\theta,\delta_{TE},l_s)$. In brief, since a segment can recombine with its counterpart on another genome, we assume that each segment has its own phylogeny, which is different from the phylogeny of the genomes; the population structure at each segment is approximated by the coalescent model. For an attempted recombination between Y and an external donor D, we can use the coalescent model to calculate the probability distribution for the segment divergence δ between D and X, and then obtain x from $x=l_s\delta$. The expression of $P(x|y,\theta,\delta_{TE},l_s)$ is:

$$P(x|y,\theta,\delta_{TE},l_s) = \frac{1}{B}[\Theta(y-x)A_1(x|y,\theta,\delta_{TE},l_s) + \Theta(x-y)A_2(x|y,\theta,\delta_{TE},l_s) + \delta_{xy}A_3(y,\theta,\delta_{TE},l_s)]$$

B is a normalization constant; $\Theta(x)$ is the step function, which is 1 when $x>0$ and 0 when $x\leq 0$; δ_{xy} is the Kronecker delta. The three terms of $P(x|y,\theta,\delta_{TE},l_s)$ (corresponding to Eq. (S1-S3) in *Dixit et al.* (Dixit et al. 2017) represent three different possible scenarios of recombination:

1. when $x<y$, recombination reduces divergence: $A_1(x|y,\theta,\delta_{TE},l_s) = \frac{1}{l_s\theta}exp(-\frac{y}{l_s\delta_{TE}})exp(-\frac{2x}{l_s\theta})$;
2. when $x>y$, recombination increases divergence: $A_2(x|y,\theta,\delta_{TE},l_s) = \frac{1}{l_s\theta}exp(-\frac{x}{l_s\delta_{TE}})exp(-\frac{y}{l_s\theta})exp(-\frac{x}{l_s\theta})$;
3. when $y=x$, the recombination event either failed, or succeeded but did not change the divergence: $A_3(y,\theta,\delta_{TE},l_s) = 1 - \sum_{x=0}^{y-1} A_1(x|y,\theta,\delta_{TE},l_s) - \sum_{x=y+1}^{l_s^{cutoff}} A_2(x|y,\theta,\delta_{TE},l_s)$.

The normalization constant *B* is introduced to deal with possible numerical errors and is determined by the condition:

$$\sum_{x=0}^{l_s^{cutoff}} P(x|y,\theta,\delta_{TE},l_s) = 1$$

We can calculate $f(x|t)$ at different *t*, starting from Equation (1) with the boundary condition $f(x|0)=\delta_{x0}$ and using discrete time step *t*. Supplementary Figure S1 shows an example of SSP distributions of this model at different coalescent time *t*, with model parameters (μ, ρ, θ, δ_{TE}, l_s)=(1E-5, 0.01, 2%, 1%, 1000).

Monte Carlo algorithm implementing the CGP model to search for the optimal tree and model configuration

We developed a Monte Carlo algorithm, along with Metropolis acceptance and annealing, to search for the parameters ($\mu,\rho,\theta,\delta_{TE}$) and coalescent time of all pairs of sequences that maximize the score in Eq. (3). Given the initial parameters ($\mu,\rho,\theta,\delta_{TE}$), the algorithm rescales time, such that after rescaling $l_s\mu$=min(0.02, $l_s\delta_{farthest}$/200); here $\delta_{farthest}$ is the sequence divergence between the most divergent pair. This rescaling rule has two considerations: (i) $l_s\mu$ (and also ρ) should be ≪1 to suppress numerical error; (ii) the branch lengths, which is the coalescent time *t* in $f(x|t)$, should be numerically small to save computational resource. The algorithm calculates the initial $f(x|t)$, discretizing time *t* into steps; the coalescent time of genome-pair XY, t_{XY}, is set to make $f(x|t)$ best-fit $g_{XY}(x)$. This generates an initial *t*-matrix of the genome-pairs, which is turned into ultrametric tree by single linkage clustering.

The algorithm maps an *n*-leaves ultrametric tree to an $n\times n$ coalescent time matrix through single linkage clustering. Moving in the direction of a degree of freedom in this tree is like moving an internal node up or down, joining two nodes or splitting one node. Hence the matrix has ≤*n*-1 degrees of freedom despite n^2 entries. When performing local search, CGP algorithm generates a new tree by moving an internal node of the old tree up or down by one time-step. This may combine two internal nodes into one; for an internal node with $c>2$ children, a possible move involves moving the entire node upwards or downwards, or

breaking it into two nodes, one with two children and the other with c-1 children (see Supplementary Figure S2 for an example).

Given the initial tree and model parameters, the algorithm proceeds to search for the optimal tree and model parameters. In each Monte Carlo step, one of the following moves is considered and selected according to Metropolis acceptance:

1. There is an $n^{-2}/2$ chance to mutate one of the parameters ($\rho, \theta, \delta_{TE}$). The algorithm considers one of six different parameter sets ($\rho(1+\varepsilon), \theta, \delta_{TE}$), ($\rho(1-\varepsilon), \theta, \delta_{TE}$), ($\rho, \theta(1+\varepsilon), \delta_{TE}$), ($\rho, \theta(1-\varepsilon), \delta_{TE}$), ($\rho, \theta, \delta_{TE}(1+\varepsilon)$), ($\rho, \theta, \delta_{TE}(1-\varepsilon)$), with random variable ε, $1 \gg \varepsilon > 0$. Absolute upper limits are imposed for some of the parameters: $\rho<1$,$\theta<100\%$ and $\delta_{TE}<100\%$.
2. There is an $n^{-2}/2$ chance for a random branch of the tree to be cut and grafted to a different part of the tree. In this move, a branch with the younger internal node Y and older internal node O is picked randomly. With their ages denoted as t_Y and t_O, this branch is then cut at the height t_O, and the entire sub-clade is then grafted to another random branch on the tree that is present at height t_O to generate a new ultrametric tree.
3. There is a $1-n^{-2}$ chance for an internal node to be picked randomly. One of the possible local moves of the node described above is selected randomly to generate a new tree.

The simulation continues, and stops when the maximal score of the chain of Monte Carlo steps has not increased by more than 1 for the last 200,000 steps. It also makes use of annealing, starting with an initial temperature that has magnitude of a twenty-thousandth of the initial score, and reducing by half every 50,000 steps.

Preparing simulated and real genome sequences to test different phylogenetic algorithms

Fisher-Wright simulation

We simulated Fisher-Wright populations that have micro and macro recombination. We set N_e=1000, genome length 5Mb, and each site can have one of the four letters ATGC. In our simulation, mutation occurs at a rate μ per site, which randomly mutates a site to one of the other three nucleotides. A recombination-attempting DNA stretch initiates at a rate ρ_{ini} per genome per time-step, which transfers a DNA stretch from a random donor that starts at a random site of the genome. The length of the stretch is drawn either from a micro distribution (geometric distribution, mean 100bp) or from a macro distribution (geometric distribution, mean 1kb), both with equal probability. The recombination attempt can either succeed (the donor stretch replaces the recipient stretch) or fail; the chance of success is $\exp(-\delta/\delta_{\mathrm{TE}})$, where δ is the divergence between the incoming stretch and the host stretch. Throughout a simulation, we recorded the history of genome inheritance and have full knowledge of the population phylogenetic tree. A simulation continues for at least 10,000 steps, and then stops once the MRCA of the population has emerged.

We performed Fisher-Wright simulation on three sets of parameters (μ, ρ, θ, δ_{TE}), representing prokaryotic species with low, intermediate and high levels of recombination; note that $\rho=\rho_{\mathrm{ini}}L$, where L is the average length of the transferred DNA stretch and is 550bp in our case. The parameters of the simulations are:

1. (5E-5, 0.01, 10%, 0.8%), r/m≈2

2. (5E-5, 0.25, 10%, 0.8%), r/m≈40
3. (2.5E-5, 0.25, 5%, 0.8%), r/m≈80

r/m is the ratio between substitutions contributed by mutations and by recombinations; these r/m values are estimated using Eq. (A2) of *Dixit et al.* (Dixit et al. 2017). We set the transfer efficiency δ_{TE}=0.8%, which is close to previously reported values 2.4% (Fraser et al. 2007) and 0.8% (Dixit et al. 2015) for *E. coli*. A previous study reports the r/m values of different prokaryotic species, which range from 0.02 to 63.6 (Didelot et al. 2009); hence the different levels of recombination of our simulated genomes are consistent with what observed in nature.

We picked genomes from the simulated populations that can mimic inter-species recombination. If we pick random genomes into a test-group, then their MRCA will have an average age t_{root}~1000 because N_e=1000. We would like to simulate the condition where a single lineage diverging from the rest of the population and forming its own subpopulation, so that the genomes in their subpopulation continue exchanging DNA among them and with the rest outside. Therefore, we need test-groups of genomes coming from a small clade of the population, and we constrained the MRCA of a genome-test-group to be small, i.e., $t_{\text{test-group-root}} \ll 1000$.

We performed Fisher-Wright simulations, repeating each parameter set ten times to get ten populations. We picked 10 test-groups in each population; each test-group has ~10 genomes and a constraint on the age of their MRCA: $t_{\text{test-group-root}}$~10, 20, …, 100; the true phylogenetic tree of the genomes in each test-group is also recorded. In sum, this generates 100 test-groups for each of the three parameter sets. See Additional File 4 for the sequences and their tree for each test-group.

A test-group with *n* genomes has *n*(*n*-1)/2 genome pairs, and we calculated the SSP distributions of these pairs to prepare for phylogenetic reconstruction using our model, with segment size l_s=150 (and l_s^{cutoff}=100); the last segment is discarded as it is shorter than the others.

Real *E. coli* genomes

We also made test-groups using different combinations of genome sequences chosen from 55 *E. coli* strains (see Supplementary Table S1 for their names and Genbank Accession IDs). We detected the orthologous gene families in these 55 genomes by performing the ProteinORTHO algorithm (v5.11) (Lechner et al. 2011) on their amino acid coding sequences (CDS), which identifies 1,636 orthologous gene families that have one copy in each of these 55 strains; see Additional File 2 for a list of these core genes and Additional File 4 for the genes to orthologous gene families mapping provided by ProteinORTHO. We aligned the nucleotide sequences of these 1,636 universal orthologous gene families using MAFFT (Katoh and Standley 2013) with options "--maxiterate 1000" and "--localpair"; we performed another alignment on their amino acid sequences in the same way.

To evaluate the accuracy of different phylogenetic algorithms using GLOOME, we made 100 test-groups, each with ten strains randomly chosen from the 55 strains. We concatenated the alignment of all universal orthologous gene families to make a 'super-gene' nucleotide sequence and a super-gene amino acid sequence for each strain (see Additional File 2 for the strains in each test-group, and Additional File 4 for their sequences). To prepare the pairwise SSP distributions, we removed all positions that have dashes in the alignment of each core gene, and divided the alignment into segments with length l_s,

ignoring the last segment if it has fewer than l_s sites. For each pair of strains in a test-group, we collected their segments in different core genes, enumerated the SSPs on each segment to get their pairwise SSP distribution; note that each pair of strains has a SNP distribution obtained from their nucleotide sequences, and a SAP distribution from their amino acid sequences. We used segment sizes l_s=150 (l_s^{cutoff}=100) for nucleotide sequences, and l_s=50 (l_s^{cutoff}=50) for amino acid sequences.

Alternatively, we evaluated the consistency of the phylogenetic algorithms by comparing pairs of phylogenetic trees reconstructed from sequences of different core genes of identical strains. We made another 100 test-groups that are different from the 100 groups in the GLOOME-test, where each group has ten strains randomly chosen from the 55 strains. In each group, we randomly assigned a core gene to be either in set A or set B. We concatenated the gene sequences in set A (B) of a strain to make its super-gene A (super-gene B); we then applied different phylogenetic algorithms to reconstruct tree A (tree B) from the super-gene A (super-gene B) of different strains in the group. For each pair of strains in a test group, we also prepared the SSP distribution A and SSP distribution B, using the nucleotide sequences (or amino acid sequences) of the genes in set A and set B, in the same way that is done in the GLOOME-test; we then reconstructed tree A and tree B using the CGP algorithm. See Additional File 4 for the strains in each test-group, the core genes of set A and set B, and also their sequences. See Additional File 3 for the unrooted SD, the unrooted BSD, and the rooted BSD between the pair of reconstructed trees in each test group.

Reconstructing the phylogeny of the simulated / real genome sequences in each test-group using RAxML, ClonalFrameML and Gubbins

Simulated genome sequences

We applied RAxML (v8.2.9) (Stamatakis 2014), ClonalFrameML (v1.11) (Didelot and Wilson 2015) and Gubbins (v2.2.0) (Croucher et al. 2015). We used the model GTRGAMMA in RAxML. We mid-point-rooted the trees reconstructed by RAxML and Gubbins using RAxML (with option '-f I'). ClonalFrameML requires a tree as input, and we fed it with the rooted RAxML tree.

Real *E. coli* genome sequences

For the nucleotide sequences, we used the algorithms and settings same as the simulated sequences. For amino acid sequences, we used RAxML with the model PROTGAMMAAUTO; the resultant trees are mid-point-rooted by RAxML (option '-f I').

Evaluating how the UPGMA assumption perturbs the coalescent time inference in CGP algorithm

To evaluate how the UPGMA assumption perturbs the coalescent time inference, we fitted our theoretical model to the SSP distributions of real genomes across the 100 GLOOME-test-groups. Based on the CGP phylogenetic reconstruction performed on the pairwise SSP distributions in each group, we obtained the optimal set of model parameters (μ, ρ, θ, δ_{TE}) and also the optimal tree that maximize the fit score. We then simulated the theoretical distribution using this optimal parameter set, and fitted it to the empirical SSP distribution of different pairs of genomes to infer their coalescent times. For a pair of genomes, we defined the directly inferred coalescent time, t_{direct}, to be the time that minimizes the cross entropy

between the theoretical distribution and the empirical distribution of the pair; alternatively, the coalescent time of the pair reported by the optimal tree is denoted as t_{tree}. To quantify the perturbation caused by UPGMA assumption, we calculated Δ, the deviation between t_{direct} and t_{tree}, defined as $\Delta = (t_{direct} - t_{tree})/(t_{direct} + t_{tree})$.

Estimating the CPU time of phylogenetic reconstruction performed by different algorithms

To assess and compare the CPU load of different phylogenetic reconstruction algorithms, we executed the linux shell script for the reconstruction of a phylogenetic tree within the Perl environment, and used the Perl package "Benchmark" to help record the time at the start and at the end of each phylogenetic reconstruction. We then applied the function "timediff" within package "Benchmark" to calculate the time difference between the start and the end, and then the function "timestr" to print out the time measurement. "timestr" outputs the "wallclock time", "user time", "system time", "user time of children", and "system time of children". The wallclock time corresponds to the time that physically elapsed between the start and the end of the script. However, not all the algorithms are executed in single-thread-mode: RAxML uses at least two threads, and Gubbins calls RAxML and thus is also multi-threaded. Therefore, we summed up the user time, system time, user time of children and system time of children, and used this sum to represent the CPU load. We performed time measurement for the phylogenetic reconstructions of the 100 GLOOME-test-groups using different algorithms, in a computer system with Linux distribution Debian GNU/Linux 10 (buster), kernel 4.19.98-1, and AMD EPYC 7601 CPU.

Evaluating the quality of a reconstructed tree

Trees reconstructed from simulated genome sequences

We measured the unrooted symmetric distance (SD) (Robinson and Foulds 1981) between a reconstructed tree and the true tree to quantify the accuracy of topology. We measured the unrooted and rooted branch score distance (BSD) (Kuhner and Felsenstein 1994) between a reconstructed tree and the true tree to quantify the branch lengths and root positioning of the reconstructed tree; before calculating the branch score distance of two trees, we normalized each tree by dividing the length of each branch by the total branch length.

Trees reconstructed from real *E. coli* sequences

We evaluated the accuracy of a reconstructed tree by comparing the tree to the phylogenetic signals encoded in the absence and presence of genes across different genomes. We considered each internal node of the tree to be an ancestral strain, and used the GLOOME algorithm (VR01.266) (Cohen et al. 2010) along with the default parameters of the online version of GLOOME (Evolutionary model: fixed gain/loss ratio, rate distribution Gamma), to reconstruct the presence and absence of different orthologous gene families in the ancestral strains. GLOOME takes a tree, and also the presence and absence of different orthologous gene families across the extant strains of the tree as input. As we have used Proteinortho (Lechner et al. 2011) to identify the orthologous gene families in the 55 *E. coli* genomes, an orthologous gene family is present in an extant strain if there is one or more copies there, and absent otherwise. The GLOOME output includes the GLOOME posterior likelihood (GPL) for the ancestral genome reconstruction. We used GPL to quantify the accuracy of a reconstructed phylogenetic tree, because the higher is the GPL, the more consistent is the

tree with the phylogeny inferred from the distribution of orthologous gene families across different extant genomes.

SUPPLEMENTARY FIGURES

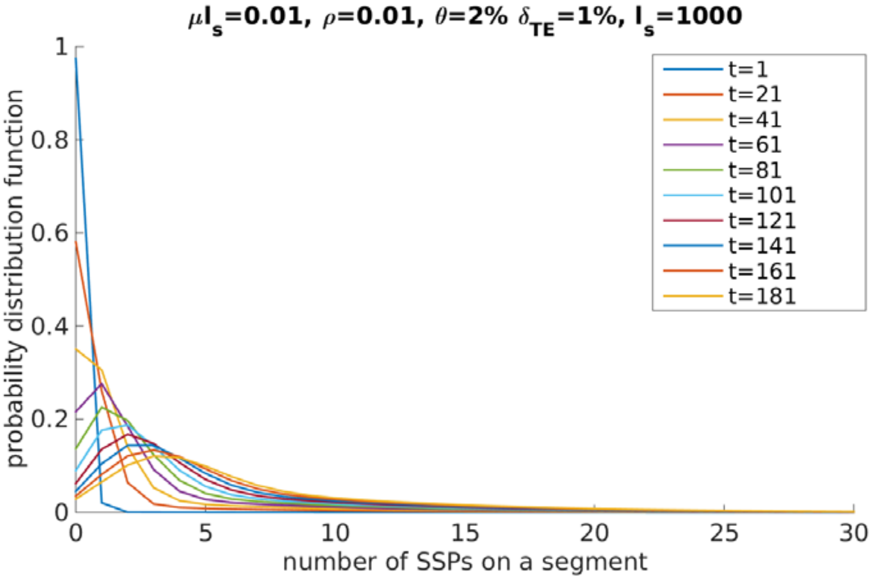

Supplementary Figure S1. SSP distribution curves at different time *t* predicted by the model (Eq. (1)), using the parameters μl_s=0.01, ρ=0.01, θ=2%, δ_{TE}=1%, l_s=1,000.

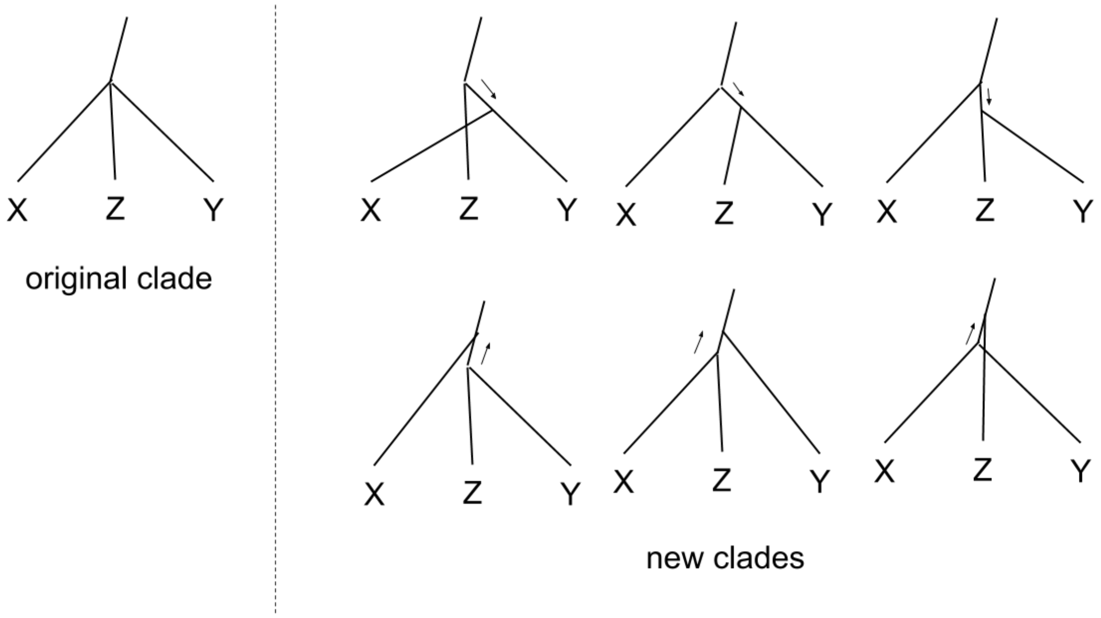

Supplementary Figure S2. Illustration of possible local moves that split an internal node into two. For an internal node that has more than two children, possible moves comprise moving the node upwards or downwards by one time-step (not shown), and also moving one of its downstream branches upwards or downwards by one time-step and splitting the internal node into two (all 6 possible moves that split an internal node with 3 children are shown).

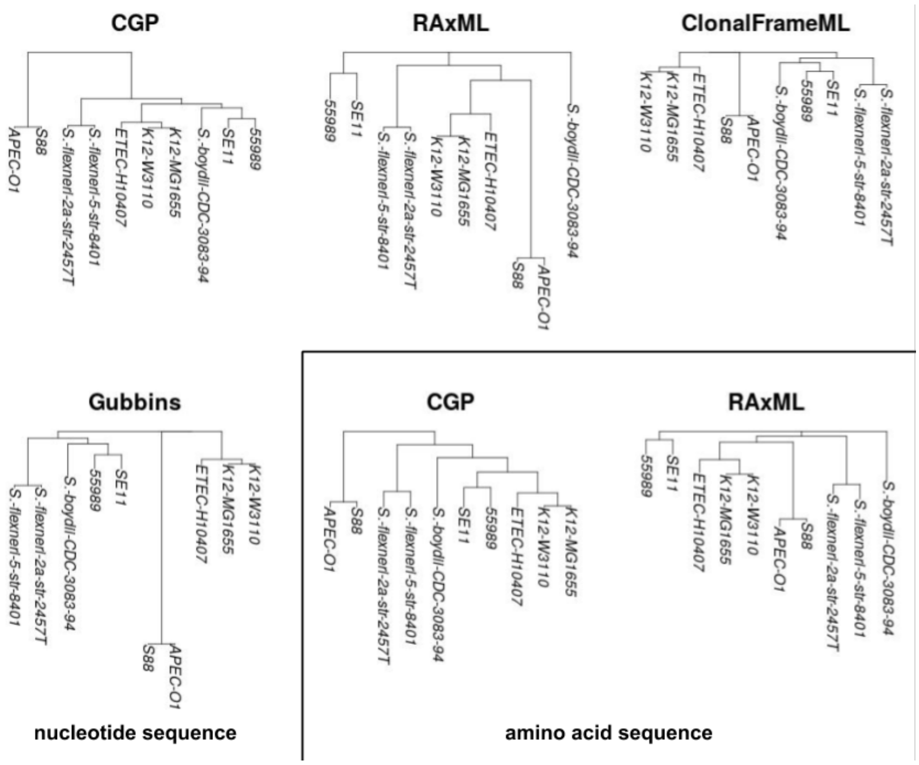

Supplementary Figure S3. An example of phylogenetic trees of 10 *E. coli* / *Shigella* strains reconstructed by CGP, RAxML, ClonalFrameML and Gubbins from the nucleotide sequences and also from the amino acid sequences. This group is one of the one hundred groups of real genome sequences prepared for the GLOOME-test.

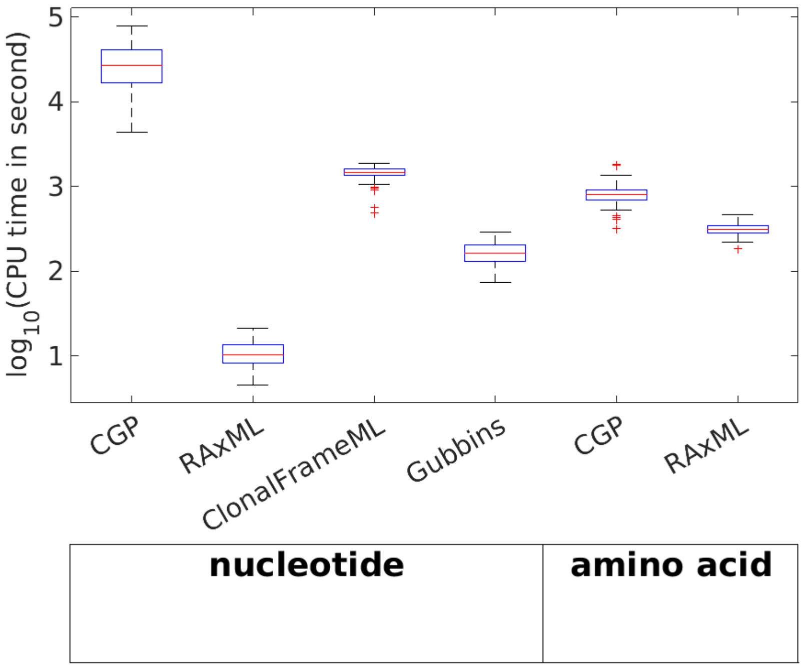

Supplementary Figure S4. Distribution of CPU time for the reconstruction of a phylogenetic tree, by CGP, RAxML, ClonalFrameML, and Gubbins applied to nucleotide sequences, and by CGP and RAxML to amino acid sequences. Note that ClonalFrameML takes the RAxML tree as input, and rescales the tree's branch lengths. See Supplementary Text for details.

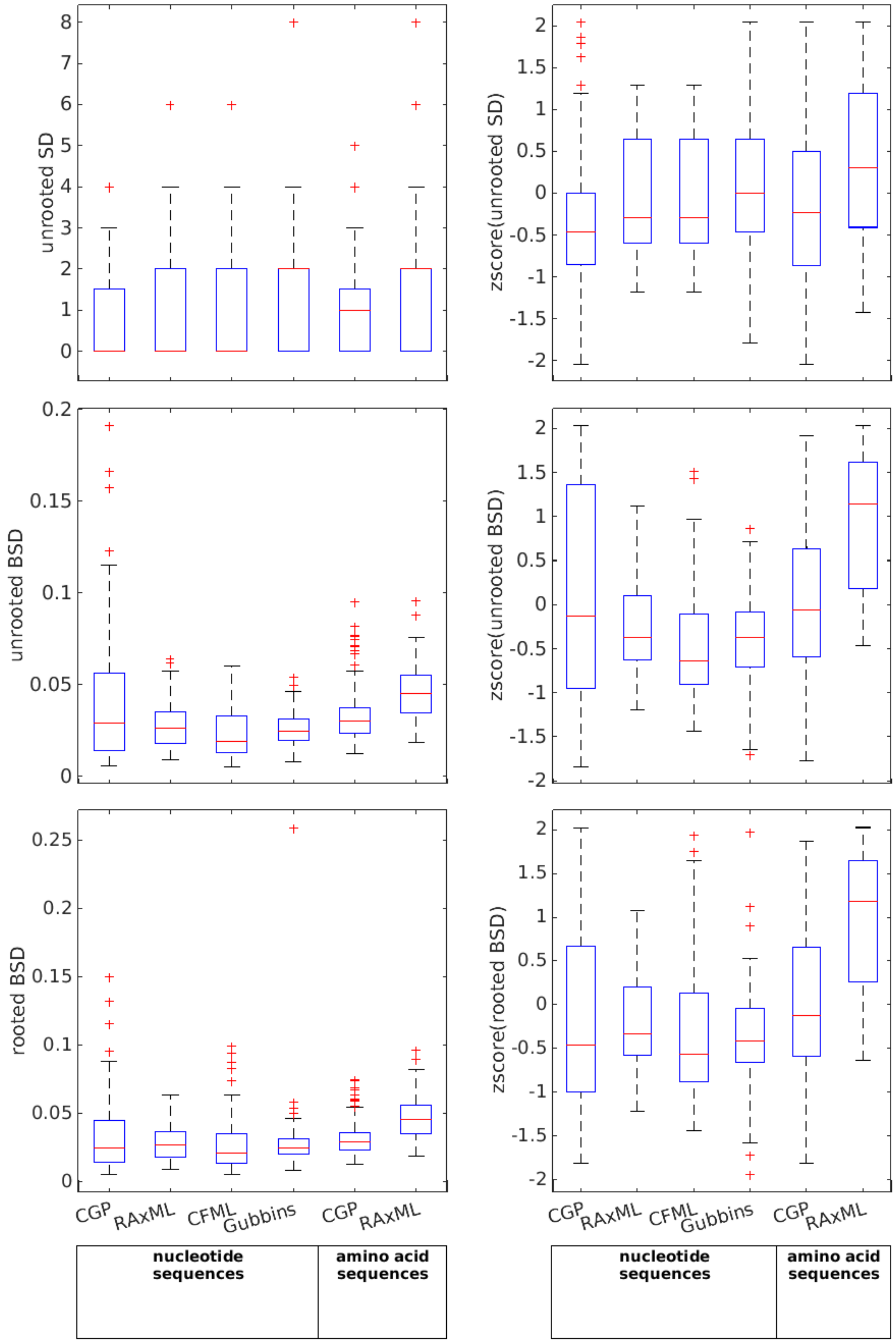

Supplementary Figure S5. Box plot showing the distribution of unrooted SD, unrooted BSD, rooted BSD, as well as the distribution of their z-score, of pairs of trees inferred from the same group of strains to evaluate the consistency of reconstruction by CGP, RAxML, ClonalFrameML, and Gubbins applied to nucleotide sequences, and also CGP and RAxML applied to amino acid sequences.

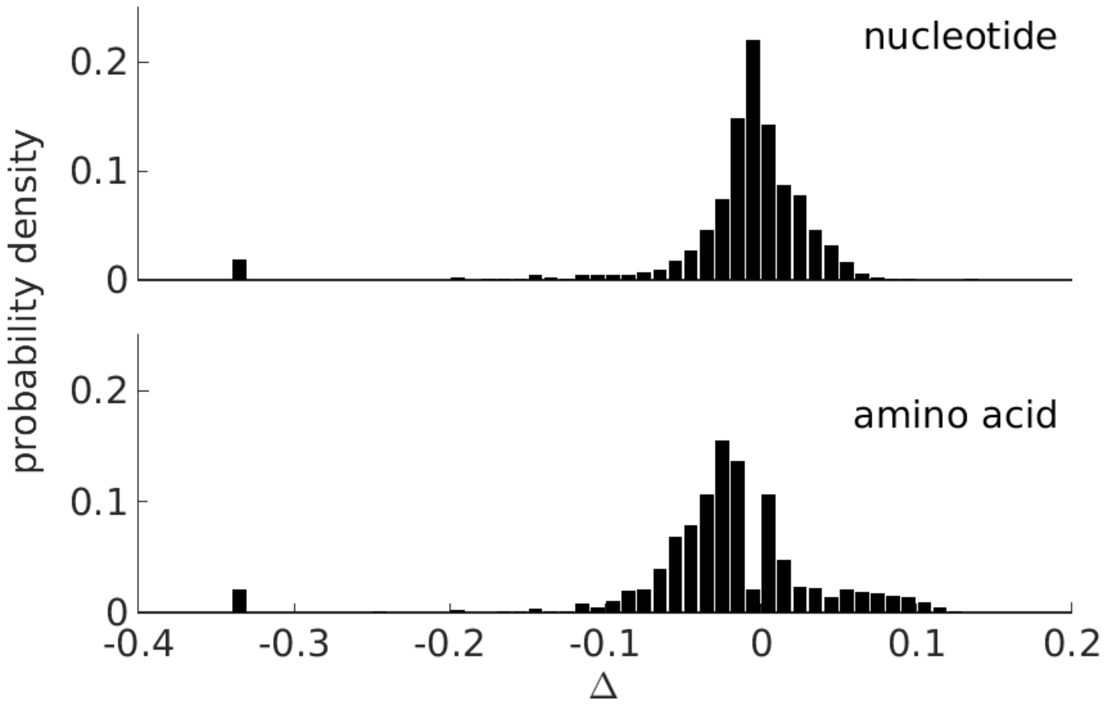

Supplementary Figure S6. Distribution of Δ—the perturbation on branch lengths / coalescent times caused by the UPGMA assumption in the CGP algorithm. See Supplementary Text for details.

SUPPLEMENTARY TABLES

Supplementary Table S1. List of 55 genomes of *E. coli* and *Shigella* used in this study.

Strain Name	Genbank Accession ID
Escherichia-coli-LF82	CU651637
Escherichia-coli-O83:H1-str.-NRG-857C	CP001855
Escherichia-coli-UM146	CP002167
Escherichia-coli-APEC-O1	CP000468
Escherichia-coli-ATCC-8739	CP000946
Escherichia-coli-B-str.-REL606	CP000819
Escherichia-coli-BL21(DE3)-BL21-Gold(DE3)pLysS-AG	CP001665
Escherichia-coli-BL21(DE3)-AM946981	AM946981
Escherichia-coli-BL21(DE3)-CP001509	CP001509
Escherichia-coli-BW2952	CP001396
Escherichia-coli-DH1	CP001637
Escherichia-coli-DH1-ME8569	AP012030
Escherichia-coli-ED1a	CU928162
Escherichia-coli-HS	CP000802
Escherichia-coli-IAI1	CU928160
Escherichia-coli-KO11FL	CP002516
Escherichia-coli-SE15	AP009378
Escherichia-coli-str.-K-12-substr.-DH10B	CP000948
Escherichia-coli-str.-K-12-substr.-MG1655	U00096
Escherichia-coli-str.-K-12-substr.-W3110	AP009048
Escherichia-coli-W-CP002185	CP002185
Escherichia-coli-W	CP002967
Escherichia-coli-042	FN554766
Escherichia-coli-O103:H2-str.-12009	AP010958
Escherichia-coli-O111:H--str.-11128	AP010960
Escherichia-coli-O157:H7-str.-EC4115	CP001164
Escherichia-coli-O157:H7-EDL933	AE005174
Escherichia-coli-O157:H7-str.-Sakai	BA000007
Escherichia-coli-O157:H7-str.-TW14359	CP001368
Escherichia-coli-O26:H11-str.-11368	AP010953
Escherichia-coli-O55:H7-str.-CB9615	CP001846
Escherichia-coli-SMS-3-5	CP000970
Escherichia-coli-O127:H6-str.-E2348/69	FM180568
Escherichia-coli-E24377A	CP000800

Escherichia-coli-ETEC-H10407	FN649414
Escherichia-coli-UMNK88	CP002729
Escherichia-coli-IHE3034	CP001969
Escherichia-coli-S88	CU928161
Escherichia-coli-536	CP000247
Escherichia-coli-ABU-83972	CP001671
Escherichia-coli-CFT073	AE014075
Escherichia-coli-IAI39	CU928164
Escherichia-coli-NA114	CP002797
Escherichia-coli-UMN026	CU928163
Escherichia-coli-UTI89	CP000243
Shigella-boydii-CDC-3083-94	CP001063
Shigella-boydii-Sb227	CP000036
Shigella-dysenteriae-Sd197	CP000034
Shigella-flexneri-2002017	CP001383
Shigella-flexneri-2a-str.-2457T	AE014073
Shigella-flexneri-2a-str.-301	AE005674
Shigella-flexneri-5-str.-8401	CP000266
Shigella-sonnei-Ss046	CP000038
Escherichia-coli-SE11	AP009240
Escherichia-coli-55989	CU928145

REFERENCES

Cohen O., Ashkenazy H., Belinky F., Huchon D., Pupko T. 2010. GLOOME: gain loss mapping engine. Bioinformatics. 26:2914–2915.

Croucher N.J., Page A.J., Connor T.R., Delaney A.J., Keane J.A., Bentley S.D., Parkhill J., Harris S.R. 2015. Rapid phylogenetic analysis of large samples of recombinant bacterial whole genome sequences using Gubbins. Nucleic Acids Res. 43:e15–e15.

Didelot X., Lawson D., Falush D. 2009. SimMLST: simulation of multi-locus sequence typing data under a neutral model. Bioinforma. Oxf. Engl. 25:1442–1444.

Didelot X., Wilson D.J. 2015. ClonalFrameML: Efficient Inference of Recombination in Whole Bacterial Genomes. PLOS Comput Biol. 11:e1004041.

Dixit P.D., Pang T.Y., Maslov S. 2017. Recombination-Driven Genome Evolution and Stability of Bacterial Species. Genetics.:genetics.300061.2017.

Dixit P.D., Pang T.Y., Studier F.W., Maslov S. 2015. Recombinant transfer in the basic genome of Escherichia coli. Proc. Natl. Acad. Sci. U. S. A. 112:9070–9075.

Fraser C., Hanage W.P., Spratt B.G. 2007. Recombination and the nature of bacterial speciation. Science. 315:476–480.

Katoh K., Standley D.M. 2013. MAFFT multiple sequence alignment software version 7: improvements in performance and usability. Mol. Biol. Evol. 30:772–780.

Kuhner M.K., Felsenstein J. 1994. A simulation comparison of phylogeny algorithms under equal and unequal evolutionary rates. Mol. Biol. Evol. 11:459–468.

Lechner M., Findeiß S., Steiner L., Marz M., Stadler P.F., Prohaska S.J. 2011. Proteinortho: Detection of (Co-)orthologs in large-scale analysis. BMC Bioinformatics. 12:1–9.

Robinson D.F., Foulds L.R. 1981. Comparison of phylogenetic trees. Math. Biosci. 53:131–147.

Stamatakis A. 2014. RAxML Version 8: A tool for Phylogenetic Analysis and Post-Analysis of Large Phylogenies. Bioinformatics.:btu033.
